# “War to the knife” against thromboinflammation to protect endothelial function of COVID-19 patients

**DOI:** 10.1186/s13054-020-03060-9

**Published:** 2020-06-19

**Authors:** Gabriele Guglielmetti, Marco Quaglia, Pier Paolo Sainaghi, Luigi Mario Castello, Rosanna Vaschetto, Mario Pirisi, Francesco Della Corte, Gian Carlo Avanzi, Piero Stratta, Vincenzo Cantaluppi

**Affiliations:** Department of Translational Medicine, School of Medicine of the University of Piemonte Orientale (UPO), Novara, Italy

**Keywords:** COVID-19, Thrombosis, Thromboinflammation, Heparin, Plasma, Endothelial dysfunction, Complement, Extracellular vesicles, Anticoagulation

## Abstract

In this viewpoint, we summarize the relevance of thromboinflammation in COVID-19 and discuss potential mechanisms of endothelial injury as a key point for the development of lung and distant organ dysfunction, with a focus on direct viral infection and cytokine-mediated injury. Entanglement between inflammation and coagulation and resistance to heparin provide a rationale to consider other therapeutic approaches in order to preserve endothelial function and limit microthrombosis, especially in severe forms. These strategies include nebulized heparin, N-acetylcysteine, plasma exchange and/or fresh frozen plasma, plasma derivatives to increase the level of endogenous anticoagulants (tissue factor pathway inhibitor, activated protein C, thrombomodulin, antithrombin), dipyridamole, complement blockers, different types of stem cells, and extracellular vesicles. An integrated therapy including these drugs has the potential to improve outcomes in COVID-19.

## Background

SARS-CoV-2 predominantly affects the lung, with possible development of acute respiratory distress syndrome (ARDS) requiring admission to intensive care units. However, several cases of multiple organ dysfunction have been reported and the pathogenic mechanisms of other organ involvement are far from being elucidated. Even though organ dysfunction may be related to low tissue oxygenation, other causes should be considered: first, a maladaptive immune response characterized by a plethora of inflammatory mediators; second, a direct viral infection of different tissues: indeed, SARS-CoV-2 enters target cells through the interaction between its spike proteins and ACE2 receptor, which is located on many types of epithelial (lung, kidney, gut) and endothelial cells. Varga and coworkers actually demonstrated the presence of SARS-CoV-2 within endothelial cells of different organs [1].

These findings suggest that endothelial cell injury may play a key role in the pathogenic mechanisms of both lung and distant organ injury. Quiescent endothelial cells maintain microvascular integrity exerting anti-inflammatory, anti-thrombotic, and vasodilatory activities. However, under pathologic conditions, endothelial cell homeostasis can be altered causing dysfunction of coagulation, complement, fibrinolysis, and kinins cascades. Even though primary efforts should be focused on drugs directly acting on SARS-CoV-2 replication, secondary mechanisms of tissue injury also represent attractive targets: among these, activation of the coagulation pathway due to endothelial injury plays a pivotal role [2].

## Main text

In a series of 26 autopsies in COVID-19 patients, the presence of a diffuse erythrocyte aggregation in renal peritubular and glomerular capillaries, with signs of endothelial activation and segmental fibrin thrombi, was observed [3]. Similar results were also found in the lungs and other organs [4]. These findings suggest that thromboinflammation is a key feature of SARS-CoV-2 infection, especially in the lungs [5, 6]. Furthermore, higher fibrin degradation product (FDP) levels, longer prothrombin time, and activated partial thromboplastin time are associated with a poor prognosis: of note, 71.4% of non-survivors met the criteria of disseminated intravascular coagulation (DIC) during their hospital stay in comparison to 0.6% of survivors [7]. Consistently, other studies showed higher D-dimer levels, thrombocytopenia, diffuse acute pulmonary embolism with normal lower-limb compression ultrasonography, acro-ischemia, skin bulla, and dry gangrene [8, 9].

For these reasons, heparin has been extensively used in COVID-19 patients [10]. Unfortunately, coagulation pathway activation remained significantly stronger in non-survivors despite heparin use, suggesting that, in this setting of explosive activation of coagulation, standard thromboprophylactic doses of subcutaneous heparin may be inadequate and therapeutic doses indicated in severe cases. Another aspect is that, apart from anticoagulant properties, anti-inflammatory effects of heparin may antagonize endothelial dysfunction [11]. Several immunomodulatory properties have been associated with unfractionated heparin (UH), which can inactivate inflammatory cytokines, inhibit neutrophil chemotaxis and leukocyte migration, neutralize complement factor C5a and sequester acute phase proteins [12]; however, these biological effects have not yet been fully elucidated for LMWH. Furthermore, the risk of hemorrhagic complications is increased, and special caution is needed not only in patients with impairment of renal function, one of the most frequent complications of COVID-19 patients, but also in those with normal renal function whenever higher therapeutic doses of heparin are needed. In these settings, monitoring of Factor Xa is recommended in order to limit hemorrhagic risk.

*Entanglement* between inflammation and coagulation and resistance to heparin opened new prospects for therapies: the *war to the knife* against thromboinflammation to protect microvascular endothelial cells in COVID-19 must rely on alternative strategies that we propose in the following 7 points:

1. Nebulized heparin has been shown to ameliorate pulmonary coagulopathy and reduce the need for mechanical ventilation in ARDS: other studies did not confirm these data, probably because of ARDS heterogeneity. However, since COVID-19-associated lung injury is characterized by diffuse microthrombosis and endothelial dysfunction, nebulized heparin could represent a potential therapeutic approach, limiting bleeding risk and increasing its effectiveness [13].
2. Taking into account the presence of systemic inflammation, *N*-acetylcysteine (e.v./oral, nebulization, or inhalation) may protect from oxidative stress-mediated endothelial damage, which activates the highly thrombotic subtype of DIC observed in COVID-19. In fact, *N*-acetylcysteine binds to glutamine and glycine generating the powerful antioxidant known as glutathione that has been shown to counteract the inflammatory response in pneumonia [14, 15].
3. In selected cases of coagulation activation and multiple organ failures, plasma exchange (PEX) could be considered. However, since PEX is not an available option for all critically ill patients in an emergency setting, high doses of fresh frozen plasma (FFP) can represent an alternative, providing factors capable of preventing fibrin formation at different levels of the coagulation cascade, with a similar mechanism to that observed in thrombotic microangiopathy [16, 17]. In these cases, indication for plasma infusion is not related to immunological reasons (administration of immunoglobulins against SARS-CoV-2) but aimed at providing natural anticoagulants and cofactors which are pathologically consumed.

4. Another interesting therapeutic option is the use of plasma derivatives capable of increasing the level of endogenous anticoagulants, such as tissue factor pathway inhibitor (TFPI), activated protein C (APC), thrombomodulin (TM), and antithrombin (AT). Since alveolar epithelium is the main source of tissue factor (TF), a major initiator of the extrinsic coagulation cascade in acute lung injury (ALI), TFPI could limit coagulation activation and cell damage. Preclinical models of ARDS showed positive results with nebulized recombinant human TFPI [18]. Similar data were obtained with nebulized APC administration in animal models of ALI, whereas e.v. administration showed negative results in patients with severe sepsis (PROWESS-Shock). ART-123 is a recombinant human soluble TM with anticoagulant and anti-inflammatory properties shown to improve outcome in patients with ARDS and DIC [19]. Furthermore, nebulized AT improved pulmonary coagulopathy and fibrinolysis in an animal septic model of ALI, without important adverse effects [20].

5. Other drugs may potentially limit endothelial dysfunction and thromboinflammation during SARS-CoV-2 infection. Dipyridamole (DIP) has been recently shown to exert a protective effect in experimental studies, as it was clinically associated with increased platelet counts and decreased D-dimer levels. Furthermore, in both in vitro and animal studies, it suppressed SARS-CoV-2 replication and promoted a type I interferon (IFN) response [21]. Recent studies suggested that the preservation of endothelial Tie2 expression protects the vasculature against thrombus formation in systemic inflammation by limiting endothelial TF expression and fibrin accumulation. In quiescent endothelial cells, angiopoietin-1 stimulates Tie2, but during inflammation, angiopoietin-2 competitively inhibits Tie2, favoring endothelial dysfunction that could be targeted using adenoviral constructs expressing the protective angiopoietin-1.

6. Another crucial mechanism in SARS-CoV-2-associated inflammatory response is the triggering of complement cascade with deposition of the final component C5b-9 and endothelial cell lysis. Therapeutically, drugs developed to block the complement system may modulate the deregulated inflammatory response in COVID-19. C3 inhibitors, such as AMY-101, already tested in humans, could have a beneficial role by early complement blockade. A specific antibody against C5a receptor (C5aR) was employed in mice infected with MERS-CoV and effectively blunted lung injury [22]. Last, Diurno et al. described a case series of 4 COVID-19 patients with severe pneumonia treated with eculizumab: of note, the authors reported improvement of clinical signs, CT scan lung lesions, and laboratory tests within 48 h of first administration [23].

7. Regenerative medicine with the use of different types of stem cells has been proposed in recent years for the cure of acute and chronic diseases. Mesenchymal stromal cells (MSC) and endothelial progenitor cells (EPC) have been studied in pre-clinical models and in clinical trials enrolling ARDS patients with promising results. EPC are mobilized from the bone marrow following vascular injury in order to induce neoangiogenesis and restore endothelial integrity. In ARDS patients, circulating EPC increase reflects microvascular damage and correlates with survival [24]. Moreover, in experimental models of acute lung injury, EPC transplantation reduced edema and hyaline membrane formation favoring endothelium repair.

On this basis, more than 30 clinical trials using MSC in COVID-19 are currently ongoing. The therapeutic potential of MSC is based on a dual beneficial effect: induction of a regenerative program in the lung epithelial and endothelial cells and simultaneous modulation of the inflammatory response (mainly by increased generation of T regulatory cells).

Of note, the protective role of stem cells is mainly due to the release of paracrine molecules including extracellular vesicles (EV). EV are microparticles that have a role in cell-to-cell communication through the transfer of proteins, membrane receptors, lipids, and genetic material such as mRNA and microRNA. Several experimental studies demonstrated the protective role of MSC- and EPC-derived EV in ARDS [25].

## Conclusions

In conclusion, we herein describe the relevance of thromboinflammation and endothelial dysfunction in COVID-19 patients. Microvascular derangement is a key mechanism of multiple organ dysfunction and therapeutic strategies other than heparin, aimed at preserving endothelial integrity, should be considered to win this *war to the knife*.

## Data Availability

Not applicable
